# Lower circulating preptin levels in male patients with osteoporosis are correlated with bone mineral density and bone formation

**DOI:** 10.1186/1471-2474-14-49

**Published:** 2013-01-31

**Authors:** Ning Li, Yi-Bo Zheng, Jie Han, Wei Liang, Jia-Yi Wang, Jie-Ru Zhou, Yi Shen, Jie Zhang

**Affiliations:** 1 Department of Rheumatology, Shanghai East Hospital, Tong Ji University, Shanghai, 200120, China; 2 Department of Geriatrics, Ningbo First People’s Hospital, Ningbo, China; 3 Department of Clinical Laboratory, Ningbo First People’s Hospital, Ningbo, China

**Keywords:** Preptin, Osteoporosis, Bone density, Bone metabolic marker

## Abstract

**Background:**

Serum preptin levels among subjects with different bone mineral densities (BMD) were measured and investigated to determine the correlation between BMD and bone-metabolic markers.

**Methods:**

Approximately 52 elderly male patients with osteoporosis, 50 elderly men with osteopaenia, and 31 age-matched normal bone mass controls participated in the study. The serum preptin levels and bone metabolic markers were measured by enzyme-linked immunosorbent assay. The relationships between preptin levels, BMD, and metabolic parameters were also assessed.

**Results:**

The serum preptin level was the lowest in the osteoporosis group and positively correlated with BMD. All the bone formation markers in the osteoporosis and osteopaenia groups were significantly reduced compared with those in the normal group. Serum preptin level was positively correlated with all the bone formation markers, whereas no correlation was observed with the bone resorption marker TRACP-5b.

**Conclusions:**

Serum preptin levels are decreased in osteoporosis and osteopaenia patients and positively correlated with BMD. Therefore, preptin is involved in the pathogenesis of osteoporosis, probably through bone formation rather than bone resorption.

## Background

Bone remodelling is essential for adult bone homoeostasis. The failure of this process often leads to the development of osteoporosis, a current major global health concern [1]. The most important factor that affects normal bone remodelling is the tightly controlled and orchestrated regulation of osteoblasts and osteoclasts. Any disturbance in this balance causes various bone diseases, including osteoporosis, which is characteristically defined as decreases bone density with high risk of fracture [2,3]. Anti-resorption agents are considered in the rebuilding of the bone remodelling balance because postmenopausal osteoporosis (type I) is characterised by bone resorption that exceeds bone formation. By contrast, the characterisation of senile osteoporosis (type II) is reduced bone formation, whereas bone resorption is nearly stable. Thus, stimulating bone formation is another way of treating osteoporosis [4].

Recent advances and increased knowledge in the regulation of osteoblastic bone development and bone mass maintenance have enhanced the current understanding on why variations in normal osteoblastogenesis regulation induces bone diseases [5,6]. Further studies on the regulatory factors promote the discovery of therapeutic targets for stimulating bone formation, and when combined with other therapies, provide the ultimate treatment for osteoporosis [7-10]. Preptin, cosecreted with insulin and amylin from β-cells [11,12], is a 34–amino acid peptide that corresponds to Asp69–Leu102 of the proinsulin-like growth factor IIE-peptide (pro-IGF-IIE) [12,13]. Preptin is a physiologic amplifier of glucose-mediated insulin secretion. Elevated plasma preptin levels in newly diagnosed patients with type 2 diabetes mellitus (T2DM) suggest that preptin participates in the pathogenesis of T2DM [14]. Preptin stimulates osteoblast proliferation and reduces osteoblast apoptosis. Preptin administration increases bone area and mineralising surface in adult mice [15]. Therefore, preptin is involved in bone anabolism and contributes to the bone mass preservation observed in hyperinsulinaemic states, such as obesity [15]. These initial results obtained in animals and human diabetes patients led us to investigate circulating preptin levels in human osteoporosis. In the present study, serum preptin levels were measured in male subjects with different bone mineral density (BMD), in order to exclude the influence of type I osteoporosis. We also assessed the association between serum preptin levels, BMD, and several metabolic parameters in these subjects.

## Methods

### Subjects

133 elderly men participated in the study. All study subjects were of Han Chinese origin and lived in the same region of the country. The subjects had no history of T2DM, no clinical evidence of any major diseases, and they were selected from the participants who had undergone routine medical check-ups from Jun 2011 to Aug 2011. None of the subjects was taking any medication that affects bone metabolism. Candidates with body mass indices (BMI) <18.5 kg/m^2^ or BMI > 25 kg/m^2^ were all excluded. Age and BMI matched control subjects were selected. This study was conducted in accordance with the declaration of Helsinki. This study was conducted with approval from the Ethics Committee of Shanghai East Hospital affiliated of Tong Ji University. Written informed consent was obtained from all participants.

### Bone mineral density (BMD)

Dual-energy X-ray absorptiometry (DXA, GE Lunar Prodigy, US) was used to determine the BMD in the anteroposterior L_2–4_ lumbar spine, left femoral neck, and left total hip of the subjects. All cases were then divided into a normal bone mass group (*n* = 31), an osteopaenia group (*n* = 50), and an osteoporosis group (*n* = 52) according to BMD (anyone of L_2-4_, left femoral neck, or left total hip).

The World Health Organization (WHO) defines osteoporosis based on the BMD measurement [16]. Osteopaenia is defined as a BMD between 1 standard deviation (SD) and 2.5 SDs below the mean for young adults (i.e., the T score), whereas osteoporosis is defined as a BMD of >2.5 SDs below the mean for young adults [12]. This definition was developed for Caucasian postmenopausal women but it appears to be valid in men as well [17].

### Enzyme-linked immunosorbent assay (ELISA)

The serum concentration of preptin was determined by ELISA (R&D, USA). The linear range of the assay was 0.3 ng/mL to 10 ng/mL. Bone formation markers [serum bone alkaline phosphatase (B-ALP), bone Gla protein (BGP), procollagen type I amino-terminal propeptide (PINP)], and bone resorption item tartrate-resistant acid phosphatase-5b (TRACP-5b) were determined by ELISA according to manufacturers’ instructions.

### Statistical analysis

The data are shown as mean ± SD. All statistical analyses were performed using the SPSS 18.0 software (SPSS Inc., Chicago, IL, USA). The baseline characteristics of the test and the control subjects were compared by one-way ANOVA, Wilcoxon rank-sum test, or chi-square test. The general linear modelling function analysis was used to control potential confounders. As our primary approach, the serum preptin concentrations were included as continuous independent variables in the multivariate models. Simple and multiple regression analyses were used to examine the association between serum preptin concentrations and the other biomarkers. All of the statistical analyses were two-sided, and a *P* < 0.05 was considered significant.

## Results

### General data

The clinical characteristics of the subjects are shown in (Table 1). No significant difference was observed between the age, body height, weight, and BMI in all three groups (*p* > 0.05). Thus, the ages, body height, weight, and BMI were all matched.

**Table 1:** Clinical characteristics of study subjects (Data are mean±SD)

**Groups**	**Cases (n)**	**Age (y)**	**Height (m)**	**Weight (kg)**	**BMI (kg/m**^**2**^**)**
Osteoporosis	52	78.07 ± 4.61	1.64 ± 0.06	59.86 ± 6.17	22.34 ± 1.84
Osteopenia	50	77.94 ± 4.51	1.64 ± 0.06	61.14 ± 5.42	22.79 ± 1.29
Normal bone mass	31	77.15 ± 3.76	1.66 ± 0.06	61.54 ± 5.00	22.42 ± 1.55

### Bone mineral density and serum preptin levels

We compared the BMD among the three groups. The L_2-4_, femur neck and total hip BMD of osteoporosis group were all lower than those of the osteopaenia group (*P* < 0.01). The same results could be seen between the osteopaenia group and the normal bone mass group (Table 2).

**Table 2:** BMD comparison among the three groups (Data are mean±SD)

**Groups**	**Cases (n)**	**L**_**2-4**_**(g/cm**^**2**^**)**	**Femur Neck (g/cm**^**2**^**)**	**Total hip (g/cm**^**2**^**)**
Osteoporosis	52	0.826 ± 0.080^##^	0. 598 ± 0.116^##^	0.698 ± 0.125^##^
Osteopenia	50	1.059 ± 0.066^##**^	0.769 ± 0.158^##**^	0.859 ± 0.154^#**^
Normal bone mass	31	1.338 ± 0.140^**^	0.896 ± 0.182^**^	0.968 ± 0.194^**^

Note: compared with the normal bone mass group, # p<0.05, ## p<0.01; compared with the osteoporosis group, ** p<0.01.

The serum preptin levels were further compared. The serum preptin level of the osteoporosis group was lower compared with that of the osteopaenia group (5.10 ng/mL ± 0.69 ng/mL vs. 7.09 ng/mL ± 1.36 ng/mL, *P* < 0.01). The serum preptin level of the osteopaenia group was lower compared with that of the normal bone mass group (7.09 ng/mL ± 1.36 ng/mL vs. 10.11 ng/mL ± 1.61 ng/mL, *P* < 0.01) (Table 3).

**Table 3:** Serum preptin levels (Data are mean±SD)

**Groups**	**Cases (n)**	**Preptin (ng/mL)**
Osteoporosis	52	5.10 ± 0.69^##^
Osteopenia	50	7.09 ± 1.36^##**^
Normal bone mass	31	10.11 ± 1.61^**^

Note: compared with the normal bone mass group, ^##^ p<0.01; compared with the osteoporosis group, ^*^ p<0.05.

**Figure 1:**
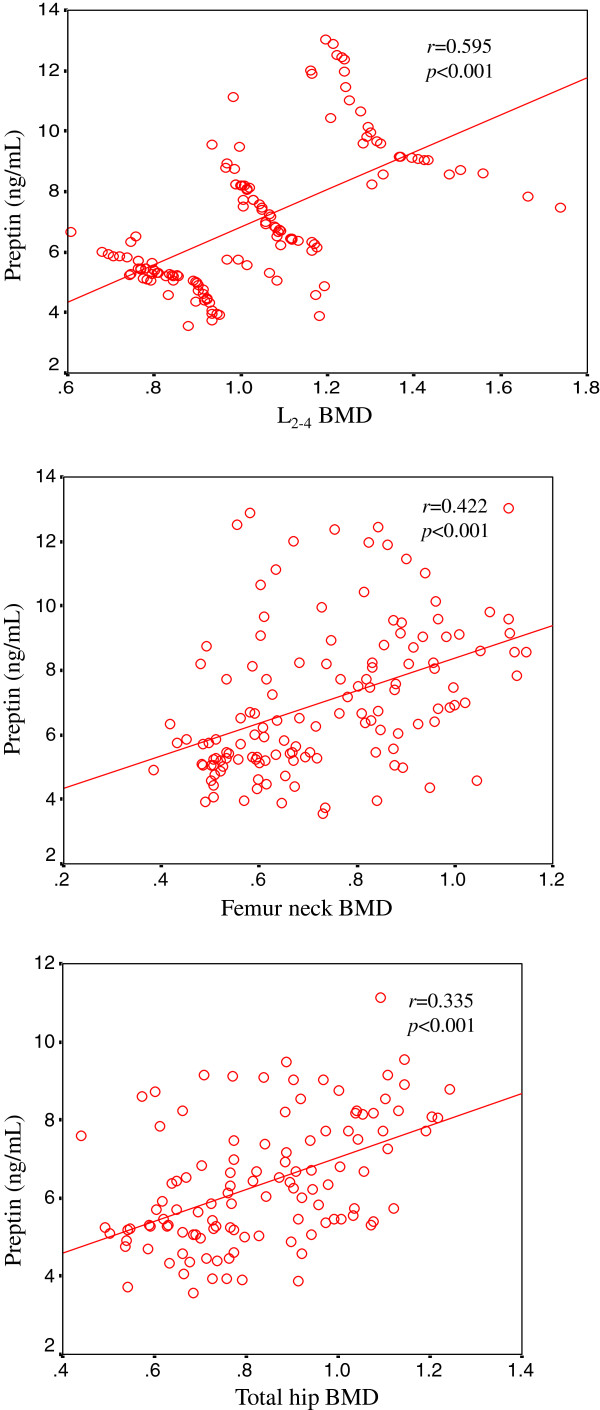
**There existed significantly positive correlation between serum preptin levels and all three BMD, that is, preptin levels correlated positively with L**_**2-4**_**BMD (*P *<0.001), femur neck BMD (*P *<0.001), and total hip BMD (*P *<0.001).**

The preptin levels correlated positively with the L_2-4_ BMD (*P* < 0.001), the femur neck BMD (*P* < 0.001), and the total hip BMD (*P* < 0.001) (Figure 1). Serum preptin levels correlated with BMD was after adjusting for age and BMI.

### Bone turnover and other biochemical parameters: correlation with preptin levels

**Figure 2:**
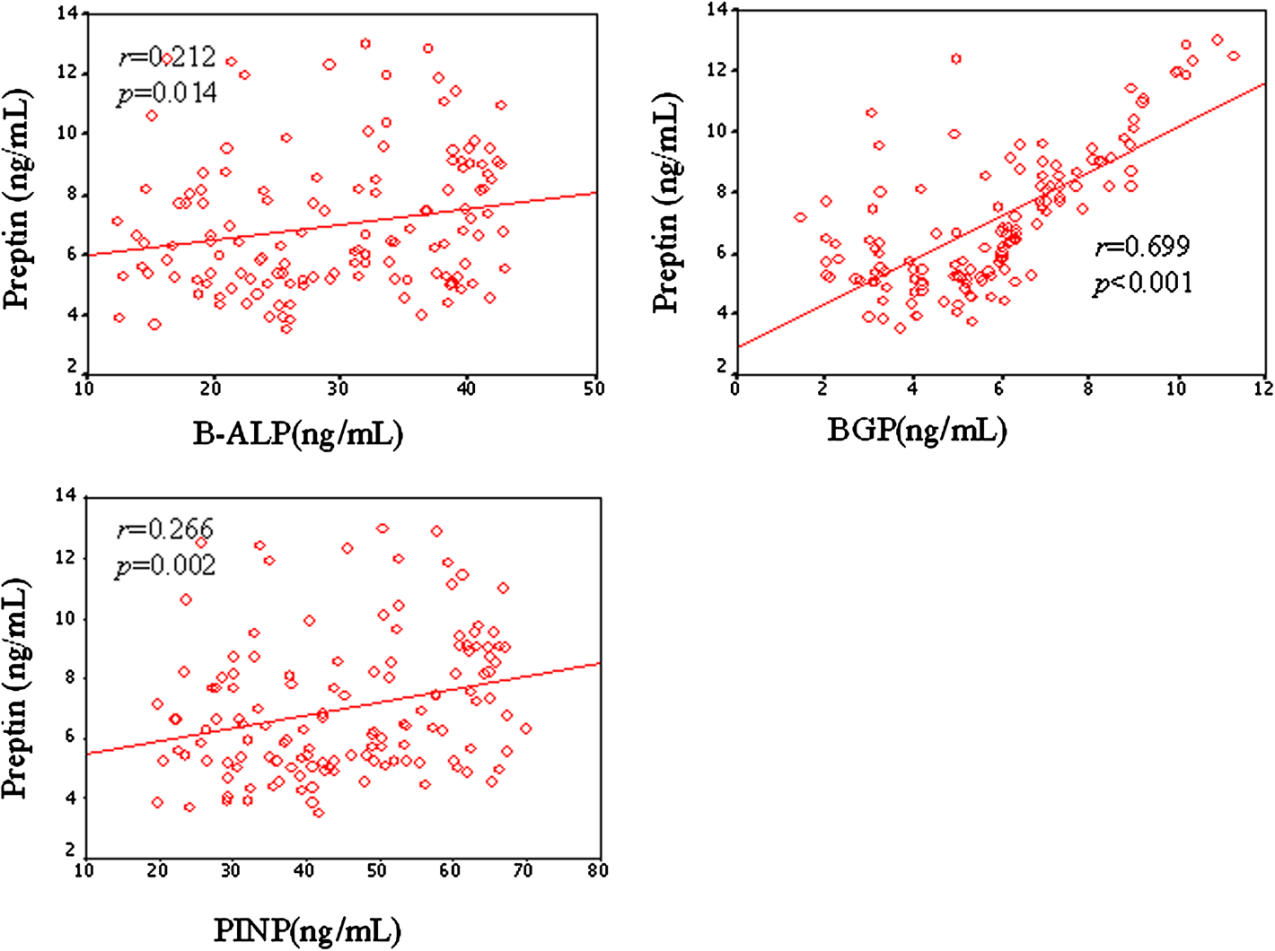
**The serum preptin levels correlated positively with all of the three bone formation items (B-ALP, p<0.05; BGP, *P *<0.001; PINP, *P *<0.01).**

The bone formation markers (B-ALP, BGP, and PINP) of the osteoporosis group were all lower than those of the osteopaenia group, and those of the osteopaenia group were lower than those in the normal bone mass group (Figure 2).

The serum preptin levels correlated positively with B-ALP (*r* = 0.212, *p* = 0.014), BGP (*r* = 0.699, *P* < 0.001), and PINP (*r* = 0.266, *p* = 0.002), which was after adjusting for age and BMI.

No significant difference was observed in the bone resorption marker TRACP-5b among the three groups (*P* > 0.05). No correlation was observed with preptin levels (*r* = −0.018, *p* = 0.841).

## Discussion

Osteoporosis is characterised by low bone mass accompanied by bone microarchitectural changes that increase fracture susceptibility [18,19]. The precise balance between bone formation and resorption is critical for the maintenance of bone mass density (BMD) and systemic mineral homoeostasis [1,2]. Osteoporosis is mainly caused by bone remodelling failure, which is regulated by many factors [20-25].

As a novel hormone, preptin increases the bone area and mineralising surface in adult mice by stimulating osteoblast proliferation and reducing osteoblast apoptosis [15]. Therefore, preptin is involved in bone anabolism and contributes to bone mass preservation [15]. However, the function and mechanism of preptin in human osteoporosis remain unclear.

The current study found that preptin levels are correlated with BMD. The preptin level was the lowest in osteoporosis patients than in osteopaenia and normal bone mass subjects. The present study is the first to investigate preptin levels in subjects with different BMDs.

Bone formation markers were reduced in elderly male patients with osteoporosis and positively correlated with preptin levels. However, the bone resorption marker was not correlated with preptin levels. Lower circulating preptin was correlated with reduced osteogenesis, which is consistent with the characteristics of Type II (senile) osteoporosis (reduced bone formation and low bone turnover). Hence, preptin may be involved in Type II osteoporosis by affecting bone formation. Understanding these mechanisms will facilitate further research on bone remodelling and osteoporosis. Future investigations on the endogenous regulation of osteoblastogenesis will increase the current knowledge for developing potential drug targets for treating osteoporosis.

The present study has limitations that need to be considered. The cross-sectional design limits our ability to infer a causal relationship between reduced serum preptin level and low BMD. Our analyses are based on a single blood preptin measurement, which does not reflect the relationship between preptin levels and BMD over time. Measuring serial changes in blood preptin levels in preosteoporotic and osteoporotic subjects will further clarify the role of preptin in the pathogenesis of osteoporosis. Furthermore, the effects of oestrogen were not included. Hence, postmenopausal osteoporotic patients were excluded from the present study. Detecting the blood preptin levels in females and comparing them with male osteoporotic patients will help elucidate the differences in pathogenesis of osteoporosis between males and females.

Diabetes mellitus (DM) alters bone remodelling, and osteopaenia and osteoporosis are among its complications. High extracellular glucose concentrations act as an endogenous factor that increases biomineralisation degree but reduces mineral quality [23]. Preptin functions in the pathogenesis of Type II DM [14]. Thus, subjects with DM or hyperglycaemia were excluded.

## Conclusion

The lower blood preptin levels in elderly male patients with osteoporosis suggest that preptin has a function in the pathogenesis of Type II osteoporosis. However, this finding needs to be clarified in further studies. Further in vivo experiments should determine whether preptin is a new target for treating osteoporosis by promoting bone formation.

## Competing interests

The authors declare that they have no competing interests.

## Authors’ contributions

All authors were involved in drafting the article or revising it critically for important intellectual content. Dr. Han J had full access to all of the data in the study and takes responsibility for the integrity of the data and the accuracy of the data analysis. Li N and Zheng Yibo were both first author of this article. Han J, Li N conceived of the study and participated in the design of the study. Li N, Zheng Yibo carried out the ELISA and analysed the data. Zheng Yibo collected the clinical data and completed the BMD analysis. Liang W performed the statistical analysis. Zhou JR, Wang JY, Shen Y, Zhang J participated in the analysis and interpretation of the data. All authors read and approved the final manuscript.

## Pre-publication history

The pre-publication history for this paper can be accessed here:

http://www.biomedcentral.com/1471-2474/14/49/prepub
